# Variants in the mitochondrial genome sequence of *Oryctes rhinoceros* (Coleoptera: Scarabaeidae) infected with *Oryctes rhinoceros nudivirus* in oil palm and coconut plantations

**DOI:** 10.1038/s41598-023-43691-w

**Published:** 2023-10-06

**Authors:** Erise Anggraini, Ganesan Vadamalai, Lih Ling Kong, Mazidah Mat, Wei Hong Lau

**Affiliations:** 1 Department of Plant Protection, Faculty of Agriculture, Universiti Putra Malaysia, 43400 UPM Serdang, Selangor Malaysia; 2 Department of Plant Pests and Diseases, Faculty of Agriculture, Universitas Sriwijaya, Indralaya, Ogan Ilir, 30662 South Sumatra Indonesia; 3 Institute of Plantation Studies, Universiti Putra Malaysia, 43400 UPM Serdang, Selangor Malaysia; 4 Malaysian Agricultural Research and Development Institute, Persiaran MARDI-UPM, 43400 Serdang, Selangor Malaysia

**Keywords:** Genetics, Zoology

## Abstract

The CRB (coconut rhinoceros beetle) haplotype was classified into CRB-S and CRB-G, based on the presence of single nucleotide polymorphisms (SNPs) in the mitochondrial *cox1* gene. Mitochondrial genomes (mitogenomes) are the most widely used genetic resources for molecular evolution, phylogenetics, and population genetics in relation to insects. This study presents the mitogenome CRB-G and CRB-S which were collected in Johor, Malaysia. The mitogenome of CRB-G collected from oil palm plantations in 2020 and 2021, and wild coconut palms in 2021 was 15,315 bp, 15,475 bp, and 17,275 bp, respectively. The CRB-S was discovered in coconut and oil palms in 2021, and its mitogenome was 15,484 bp and 17,142 bp, respectively. All the mitogenomes have 37 genes with more than 99% nucleotide sequence homology, except the CRB-G haplotype collected from oil palm in 2021 with 89.24% nucleotide sequence homology. The mitogenome of Johor CRBs was variable in the natural population due to its elevated mutation rate. Substitutions and indels in *cox1, cox2, nad2* and *atp6* genes were able to distinguish the Johor CRBs into two haplotypes. The mitogenome data generated in the present study may provide baseline information to study the infection and relationship between the two haplotypes of Johor CRB and OrNV in the field. This study is the first report on the mitogenomes of mixed haplotypes of CRB in the field.

## Introduction

*Oryctes rhinoceros* (L.) (Coleoptera: Scarabaeidae: Dynastinae), known as the coconut rhinoceros beetle (CRB), is a severe agricultural pest found in coconut and other palm trees throughout Asia and the South Pacific. CRB is an endemic insect pest of coconuts in Asia, ranging from West Pakistan to India, Ceylon, Burma, Hainan, Hong Kong, Formosa, Peninsular Malaysia, Indonesia, and the Philippines^1^, and the Pacific Islands^2^. *Oryctes rhinoceros nudivirus* (OrNV) is an endemic entomopathogenic virus affecting both the adults and immature stages of the CRB^3^. It was found in Malaysia in 1963 and introduced to the Pacific Islands to suppress the population of CRB^2^. However, OrNV has been reported failure to control the new invasive CRB in Guam (2007), Papua New Guinea (2009), Hawaii (2013), the Solomon Islands (2015), and more recently in New Caledonia and Vanuatu^4^. The intolerant of CRB to OrNV infection could be haplotype dependent^5^.

The CRB was classified into two haplotypes, CRB-S and CRB-G, based on the presence of single nucleotide polymorphisms (SNPs) in the mitochondrial *cox1* gene in 2017^5^. The haplotype CRB-S was susceptible to OrNV^5^, while the haplotype CRB-G was tolerant to OrNV. Later, CRB-PNG haplotype was identified in Fiji, Samoa, Papua New Guinea, Tonga, and the Solomon Islands in 2021^6^. The susceptibility of CRB-PNG towards OrNV infection is not reported. Different haplotypes of CRB vary in their tolerance to OrNV infection. Mixture of CRB haplotypes in the field may affect the successful use of OrNV as a biological control measure in controlling the CRB in the field.

The *cox1* gene and several other mitochondrial genes are routinely used as a universal barcoding region to identify CRB^5,7^. The presence of a single SNPs found in the partial *cox1* gene amplicon has been used to determine the CRB-G haplotype in the Pacific Islands in the early 1900s^5,6^. However, such partial sequencing data may be challenging to distinguish the true mitochondrial lineages. The insect mitogenomes are widely used for investigating insect health, comparative and evolutionary genomics^8–10^, and molecular evolution studies due to the features of maternal inheritance^7,11–17^. The first mitogenome of CRB was reported in the Solomon Islands and has confirmed the CRB which was tolerant to OrNV infection was CRB-G haplotype^5,7^. To date, the mitogenome of CRB-S with susceptibility to OrNV infection and CRB-PNG with unclear pathogenicity has yet been reported.

CRB has long been reported to infest oil palm and coconut trees in Malaysia. Johor is the second largest oil palm planted area^18^ and the largest coconut planted area in Peninsular Malaysia^19^. Four types of OrNV have been detected in the local CRB^20^. The type A OrNV was detected in many places in Malaysia while the type B OrNV was detected in Selangor, Perak and Johor. Type C and D were localized OrNV in Sabah and Kelantan, respectively. Although a high incidence of CRB infestation was reported in Johor^21^, no research has been conducted to reveal the haplotype of CRB and their interaction with OrNV in the field. This paper reports the mitogenome of CRB haplotypes and their OrNV incidence in the oil palm and coconut palms in Johor, Malaysia. This comparative mitogenome study could aid in the biosecurity and control effort against this invasive pest in Malaysia.

## Results

### Mitochondrial genome assembly

The mitochondrial genomes of Johor CRB were successfully assembled. The Johor CRB from oil palm and coconut plantations contained different sizes of mitogenomes (Table 1). Two groups of mitogenomes were found in the oil palm and coconut CRBs. The first group consists of Johor CRB (oil palm Johor CRB 2020, oil palm Johor CRB 2021 and coconut Johor CRB 2021) with mitogenome size approximately 15,315 bp to 15,484 bp while the second group consists of Johor CRB (oil palm Johor CRB 2021 and coconut Johor CRB 2021) with mitogenome size around 17,200 bp. Among the Johor CRBs examined, the smallest mitogenome (15,315 bp) was recorded in the oil palm Johor CRB 2020 (ON764799) while the biggest mitogenome (17,275 bp) was found in the coconut Johor CRB 2021 (ON764801).

**Table 1:** Mitochondrial genome statistics of CRBs collected from Johor, Malaysia.

Sample ID	Sample name	Genome size (bp)	Contig	% GC	% A	% C	% G	% T
ON764799	Oil palm Johor CRB 2020	15,315	1	28.8	39.5	18.8	10.0	31.7
ON764800	Oil palm Johor CRB 2021	15,475	1	33.9	35.8	21.7	12.3	30.3
OP694176	Oil palm Johor CRB 2021	17,142	1	28.5	39.3	18.6	9.9	32.3
ON764801	Coconut Johor CRB 2021	17,275	1	28.5	39.3	18.6	9.9	32.2
OP694175	Coconut Johor CRB 2021	15,484	1	29.0	39.2	18.9	10.1	31.8

The mitogenome of Johor CRBs was high A + T bias. Among the Johor CRB with smaller mitogenomes, oil palm Johor CRB 2020 (ON764799) and coconut Johor CRB 2021 revealed similar range of A, C, G, and T content. Approximately 250 × coverage was recorded in the mitogenome of oil palm CRB 2020 (ON764799) containing 39.5% A, 18.8% C, 10.0% G, and 31.7% T. The mitogenome of coconut Johor CRB (OP694175) contained 39.2% A, 18.9% C, 10.1% G and 31.8% T with 840 × coverage. The mitogenome of the oil palm Johor CRB collected in 2021 (ON764800) was slightly bigger than those collected in 2020, which was 15,475 bp (approximately 265 × coverage) with 35.8% A, 21.7% C, 12.3% G, and 30.3% T. The second group of Johor CRB with bigger mitogenome contained similar A (39.3%), C (18.6%), G (9.9%) and T (32.2–32.3%) content.

### Mitochondrial genome annotation

The mitogenome of Johor CRBs contained 13 protein-coding genes (PCGs), two ribosomal RNA genes, and 22 transfer RNA genes (Tables 2, 3, 4, 5 and 6). All PCGs started with a regular initiation codon (ATN). A total of 10 out of 13 PCGs had conventional stop codons (TAG or TAA) while three other genes, such as *atp6*, *cox3*, and *nad5*, had an incomplete stop codon (TAT). The annotation of all mitogenomes revealed 37 genes, with the *trnI* and *trnQ* genes rearranged in the following order: control region-*trnQ*-*trnI*-*trnM*-*nad2* instead of control region-*trnI*-*trnQ*-*trnM*-*nad2* in invertebrates.

**Table 2:** Mitogenome annotation of oil palm Johor CRB-G 2020.

Feature name	NCBI feature key	Minimum	Maximum	Length	Direction	Start codon	Stop codon
trnQ	tRNA	101	169	69	Reverse		
trnI	tRNA	227	290	64	Forward		
trnM	tRNA	295	363	69	Forward		
nad2	Gene	364	1371	1008	Forward	ATT	TAA
trnW	tRNA	1370	1435	66	Forward		
trnC	tRNA	1428	1492	65	Reverse		
trnY	tRNA	1493	1556	64	Reverse		
cox1	Gene	1558	3088	1531	Forward	ATC	TAA
trnL2	tRNA	3089	3154	66	Forward		
cox2	Gene	3155	3842	688	Forward	ATA	TAA
trnK	tRNA	3843	3913	71	Forward		
trnD	tRNA	3913	3975	63	Forward		
atp8	Gene	3976	4131	156	Forward	ATT	TAA
atp6	Gene	4125	4794	670	Forward	ATG	TAT
cox3	Gene	4795	5582	788	Forward	ATG	TAT
trnG	tRNA	5583	5652	70	Forward		
nad3	Gene	5652	5999	348	Forward	ATC	TAG
trnA	tRNA	5998	6063	66	Forward		
trnR	tRNA	6063	6127	65	Forward		
trnN	tRNA	6128	6192	65	Forward		
trnS1	tRNA	6193	6259	67	Forward		
trnE	tRNA	6261	6324	64	Forward		
trnF	tRNA	6323	6388	66	Reverse		
nad5	Gene	6389	8102	1714	Reverse	ATT	TAT
trnH	tRNA	8103	8166	64	Reverse		
nad4	Gene	8166	9503	1338	Reverse	ATG	TAA
nad4L	Gene	9497	9787	291	Reverse	ATG	TAA
trnT	tRNA	9790	9854	65	Forward		
trnP	tRNA	9855	9919	65	Reverse		
nad6	Gene	9921	10,421	501	Forward	ATC	TAA
cob	Gene	10,421	11,563	1143	Forward	ATG	TAG
trnS2	tRNA	11,562	11,627	66	Forward		
nad1	Gene	11,647	12,597	951	Reverse	ATT	TAA
trnL1	tRNA	12,599	12,661	63	Reverse		
16S rRNA	rRNA	12,639	13,944	1306	Reverse		
trnV	tRNA	13,943	14,012	70	Reverse		
12S rRNA	rRNA	14,012	14,798	787	Reverse		
Control region	Misc_feature	14,862	14,996	135	None

**Table 3:** Mitogenome annotation of oil palm Johor CRB-G 2021.

Feature name	NCBI feature key	Minimum	Maximum	Length	Direction	Start codon	Stop codon
trnQ	tRNA	75	143	69	Reverse		
trnI	tRNA	201	264	64	Forward		
trnM	tRNA	269	337	69	Forward		
nad2	Gene	338	1345	1008	Forward	ATT	TAA
trnW	tRNA	1344	1409	66	Forward		
trnC	tRNA	1402	1466	65	Reverse		
trnY	tRNA	1467	1530	64	Reverse		
cox1	Gene	1532	3061	1530	Forward	ATC	TAA
trnL2	tRNA	3063	3128	66	Forward		
cox2	Gene	3129	3815	687	Forward	ATA	TAA
trnK	tRNA	3817	3887	71	Forward		
trnD	tRNA	3887	3949	63	Forward		
atp8	Gene	3950	4102	153	Forward	ATT	TAA
atp6	Gene	4099	4767	669	Forward	ATG	TAT
cox3	Gene	4769	5554	786	Forward	ATG	TAT
trnG	tRNA	5556	5625	70	Forward		
nad3	Gene	5626	5973	348	Forward	ATC	TAG
trnA	tRNA	5972	6037	66	Forward		
trnR	tRNA	6037	6101	65	Forward		
trnN	tRNA	6102	6166	65	Forward		
trnS1	tRNA	6167	6233	67	Forward		
trnE	tRNA	6235	6300	66	Forward		
trnF	tRNA	6299	6364	66	Reverse		
nad5	Gene	6366	8078	1713	Reverse	ATT	TAT
trnH	tRNA	8079	8142	64	Reverse		
nad4	Gene	8145	9479	1335	Reverse	ATA	TAA
nad4L	Gene	9476	9763	288	Reverse	ATA	TAG
trnT	tRNA	9766	9830	65	Forward		
trnP	tRNA	9831	9895	65	Reverse		
nad6	Gene	9897	10,397	501	Forward	ATC	TGA
cob	Gene	10,397	11,539	1143	Forward	ATG	TGA
trnS2	tRNA	11,538	11,603	66	Forward		
nad1	Gene	11,623	12,573	951	Reverse	ATT	TAA
trnL1	tRNA	12,575	12,637	63	Reverse		
16S rRNA	rRNA	12,638	13,919	1282	Reverse		
trnV	tRNA	13,918	13,987	70	Reverse		
12S rRNA	rRNA	13,987	14,772	786	Reverse		
Control region	Misc_feature	14,836	14,985	150	None

**Table 4:** Mitogenome annotation of oil palm Johor CRB-S 2021.

Feature name	NCBI feature key	Minimum	Maximum	Length	Direction	Start codon	Stop codon
trnQ	tRNA	1861	1929	69	Reverse		
trnI	tRNA	1987	2050	64	Forward		
trnM	tRNA	2055	2123	69	Forward		
nad2	Gene	2124	3131	1008	Forward	ATT	TAA
trnW	tRNA	3130	3195	66	Forward		
trnC	tRNA	3188	3252	65	Reverse		
trnY	tRNA	3253	3316	64	Reverse		
cox1	Gene	3318	4853	1536	Forward	ATC	TAA
trnL2	tRNA	4849	4914	66	Forward		
cox2	Gene	4915	5622	708	Forward	ATA	TAA
trnK	tRNA	5603	5672	70	Forward		
trnD	tRNA	5673	5735	63	Forward		
atp8	Gene	5736	5891	156	Forward	ATT	TAA
atp6	Gene	5888	6555	668	Forward	ATA	TAT
cox3	Gene	6555	7342	788	Forward	ATG	TAT
trnG	tRNA	7342	7405	64	Forward		
nad3	Gene	7406	7759	354	Forward	ATC	TAG
trnA	tRNA	7758	7822	65	Forward		
trnR	tRNA	7823	7887	65	Forward		
trnN	tRNA	7888	7952	65	Forward		
trnS1	tRNA	7953	8019	67	Forward		
trnE	tRNA	8021	8086	66	Forward		
trnF	tRNA	8085	8150	66	Reverse		
nad5	Gene	8157	9864	1708	Reverse	ATT	TAT
trnH	tRNA	9865	9928	64	Reverse		
nad4	Gene	9928	11,265	1338	Reverse	ATG	TAA
nad4L	Gene	11,259	11,549	291	Reverse	ATG	TAA
trnT	tRNA	11,552	11,616	65	Forward		
trnP	tRNA	11,617	11,681	65	Reverse		
nad6	Gene	11,683	12,183	501	Forward	ATC	TAA
cob	Gene	12,183	13,325	1143	Forward	ATG	TAG
trnS2	tRNA	13,324	13,389	66	Forward		
nad1	Gene	13,409	14,359	951	Reverse	ATT	TAA
trnL1	tRNA	14,361	14,423	63	Reverse		
rrnL rRNA	rRNA	14,381	15,739	1359	Reverse		
trnV	tRNA	15,704	15,773	70	Reverse		
rrnS rRNA	rRNA	15,773	16,558	786	Reverse		
Control region	Misc_feature	16,579	17,142	564	None

**Table 5:** Mitogenome annotation of coconut Johor CRB-G 2021.

Feature name	NCBI feature key	Minimum	Maximum	Length	Direction	Start codon	Stop codon
trnQ	tRNA	1683	1751	69	Reverse		
trnI	tRNA	1809	1872	64	Forward		
trnM	tRNA	1877	1945	69	Forward		
nad2	Gene	1946	2953	1008	Forward	ATT	TAA
trnW	tRNA	2952	3017	66	Forward		
trnC	tRNA	3010	3074	65	Reverse		
trnY	tRNA	3075	3138	64	Reverse		
cox1	Gene	3140	4669	1530	Forward	ATC	TAA
trnL2	tRNA	4671	4736	66	Forward		
cox2	Gene	4737	5423	687	Forward	ATA	TAA
trnK	tRNA	5425	5494	70	Forward		
trnD	tRNA	5495	5557	63	Forward		
atp8	Gene	5558	5710	153	Forward	ATT	TAA
atp6	Gene	5707	6375	669	Forward	ATG	TAT
cox3	Gene	6377	7162	786	Forward	ATG	TAT
trnG	tRNA	7164	7227	64	Forward		
nad3	Gene	7228	7578	351	Forward	ATC	TAG
trnA	tRNA	7580	7645	66	Forward		
trnR	tRNA	7645	7709	65	Forward		
trnN	tRNA	7710	7774	65	Forward		
trnS1	tRNA	7775	7841	67	Forward		
trnE	tRNA	7843	7906	64	Forward		
trnF	tRNA	7905	7970	66	Reverse		
nad5	Gene	7972	9684	1713	Reverse	ATT	TAT
trnH	tRNA	9685	9748	64	Reverse		
nad4	Gene	9751	11,082	1332	Reverse	ATA	TAA
nad4L	Gene	11,082	11,369	288	Reverse	ATG	TAA
trnT	tRNA	11,372	11,436	65	Forward		
trnP	tRNA	11,437	11,501	65	Reverse		
nad6	Gene	11,503	12,000	498	Forward	ATC	TAA
cob	Gene	12,003	13,142	1140	Forward	ATG	TAG
trnS2	tRNA	13,144	13,209	66	Forward		
nad1	Gene	13,232	14,179	948	Reverse	ATT	TAA
trnL1	tRNA	14,181	14,243	63	Reverse		
16S rRNA	rRNA	14,241	15,526	1286	Reverse		
trnV	tRNA	15,525	15,594	70	Reverse		
12S rRNA	rRNA	15,594	16,380	787	Reverse		
Control region	Misc_feature	16,444	16,578	135	None

**Table 6:** Mitogenome annotation of coconut Johor CRB-S 2021.

Feature name	NCBI feature key	Minimum	Maximum	Length	Direction	Start codon	Stop codon
trnQ	tRNA	145	213	69	Reverse		
trnI	tRNA	271	334	64	Forward		
trnM	tRNA	339	407	69	Forward		
nad2	Gene	408	1415	1008	Forward	ATT	TAA
trnW	tRNA	1414	1479	66	Forward		
trnC	tRNA	1472	1536	65	Reverse		
trnY	tRNA	1537	1600	64	Reverse		
cox1	gene	1602	3137	1536	Forward	ATC	TAA
trnL2	tRNA	3133	3198	66	Forward		
cox2	Gene	3199	3906	708	Forward	ATA	TAA
trnK	tRNA	3887	3956	70	Forward		
trnD	tRNA	3957	4019	63	Forward		
atp8	Gene	4020	4175	156	Forward	ATT	TAA
atp6	Gene	4172	4839	668	Forward	ATA	TAT
cox3	Gene	4839	5626	788	Forward	ATG	TAT
trnG	tRNA	5626	5689	64	Forward		
nad3	Gene	5690	6043	354	Forward	ATC	TAG
trnA	tRNA	6042	6106	65	Forward		
trnR	tRNA	6107	6171	65	Forward		
trnN	tRNA	6172	6236	65	Forward		
trnS1	tRNA	6237	6303	67	Forward		
trnE	tRNA	6305	6370	66	Forward		
trnF	tRNA	6369	6434	66	Reverse		
nad5	Gene	6441	8148	1708	Reverse	ATT	TAT
trnH	tRNA	8149	8212	64	Reverse		
nad4	Gene	8212	9549	1338	Reverse	ATG	TAA
nad4L	Gene	9543	9833	291	Reverse	ATG	TAA
trnT	tRNA	9836	9900	65	Forward		
trnP	tRNA	9901	9965	65	Reverse		
nad6	Gene	9967	10,467	501	Forward	ATC	TAA
cob	Gene	10,467	11,609	1143	Forward	ATG	TAG
trnS2	tRNA	11,608	11,673	66	Forward		
nad1	Gene	11,693	12,643	951	Reverse	ATT	TAA
trnL1	tRNA	12,645	12,707	63	Reverse		
rrnL rRNA	rRNA	12,665	14,023	1359	Reverse		
trnV	tRNA	13,988	14,057	70	Reverse		
rrnS rRNA	rRNA	14,057	14,842	786	Reverse		
Control region	Misc_feature	14,845	15,264	420	None		

The haplotypes of Johor CRB were confirmed as CRB-G and CRB-S by in silico digestion. The Johor CRB-G had generated three fragments (253 bp, 138 bp, and 92 bp) while the Johor CRB-S generated four fragments (181 bp, 138 bp, 92 bp, and 72 bp). The Johor CRB from oil palm and coconut plantations contained both haplotypes G and S.

The mitogenome of oil palm Johor CRB-G 2020 (GenBank accession number: ON764799) revealed one substitution in the *nad1* gene while the mitogenome of oil palm Johor CRB-G 2021 (GenBank accession number: ON764800) contained many substitutions in *nad5, nad4, nad4L, nad6, cob*, and *nad1* genes (Table 7). The mitogenome of coconut Johor CRB-G 2021 (GenBank accession number: ON764801) had no substitution nor indel. The coconut Johor CRB-S 2021 (GenBank accession number: OP694175) and oil palm Johor CRB-S 2021 (GenBank accession number: OP694176) had substitution and indels found in *nad2, cox1, cox2, atp6, nad5, nad4, nad4L, nad6, cob* and *nad1* genes.

**Table 7:** Protein-coding genes of the mitogenomes of Johor CRBs.

Mitogenome ID (GenBank accession number)	Nucleotide change	Protein-coding gene												
		*nad2*	*cox1*	*cox2*	*atp8*	*atp6*	*cox3*	*nad3*	*nad5*	*nad4*	*nad4L*	*nad6*	*cob*	*nad1*
Oil palm Johor CRB-G 2020 (ON764799) OrNV: + Symptom: +	Insertion													
	Deletion													
	Substitution													1
Oil palm Johor CRB-G 2021 (ON764800) OrNV: + Symptom: +	Insertion								6					
	Deletion								6					
	Substitution								438	346	71	140	290	253
Oil palm Johor CRB-S 2021 (OP694176) OrNV: – Symptom: –	Insertion													
	Deletion													
	Substitution	6	4	1		3			1	2	1		5	2
Coconut Johor CRB-G 2021 (ON764801) OrNV: + Symptom: –	Insertion													
	Deletion													
	Substitution													
Coconut Johor CRB-S 2021 (OP694175) OrNV: - Symptom: –	Insertion													
	Deletion													
	Substitution	6	4	1		3			1	3	1		5	2

Comparison of the 13 PCGs of the Johor CRB mitogenome with MT457815 *O. rhinoceros* isolate S4 and MW632131 *O. rhinoceros* voucher 20LW12002 using mauve alignment.

**Figure 1:**
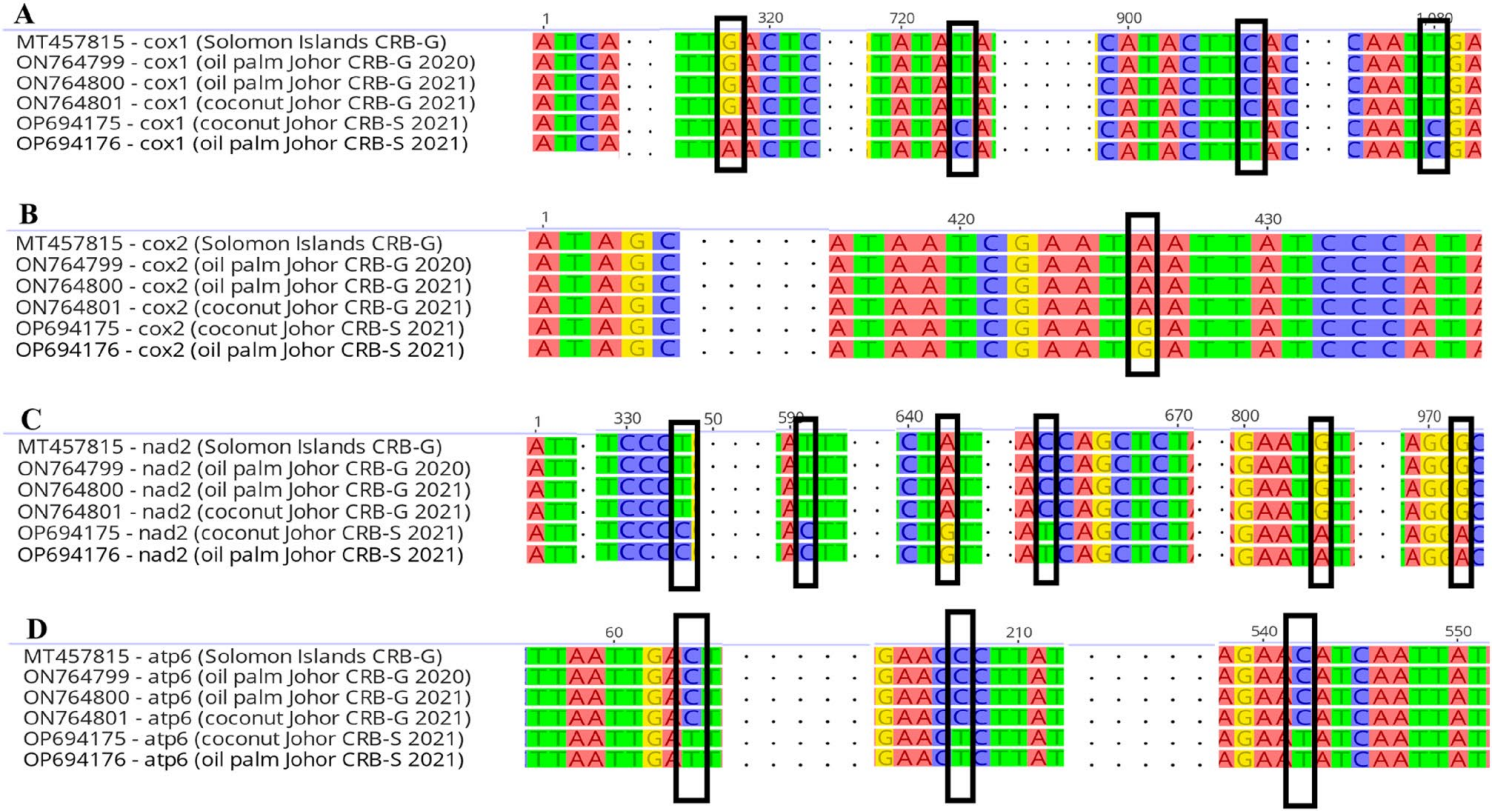
SNP locations found in (**A**) *cox1,* (**B**) *cox2,* (**C**) *nad2* and (**D**) *atp6* genes of Johor CRB mitogenomes.

A total of 13 PCGs are presented in Table 8. SNPs were detected in *cox1*, *cox2, atp6* and *nad2* genes using muscle alignment plug in Geneious Prime version 2023.0 (Fig. 1). A total of 4 SNPs was detected in *cox1* gene, 1 SNP in *cox2* gene, 3 SNPs in *atp6* gene, and 6 SNPs in *nad2* gene. The SNPs presented in Fig. 1 could differentiate the CRB-G and CRB-S significantly.

**Table 8:** Identity of based on mitochondrial genome sequences.

Specimen	Accession number	Query cover (%)	E-value	Sequence homology (%)
Oil palm Johor CRB-G 2020	ON764799	100	0	99.87%
Oil palm Johor CRB-G 2021	ON764800	100	0	89.24%
Oil palm Johor CRB-S 2021	OP694176	100	0	99.63%
Coconut Johor CRB-G 2021	ON764801	100	0	99.87%
Coconut Johor CRB-S 2021	OP694175	100	0	99.63%

The sequence homology was compared to the mitogenome of *O. rhinoceros* voucher Solomon Islands.

### Mitochondrial genome visualization

**Figure 2:**
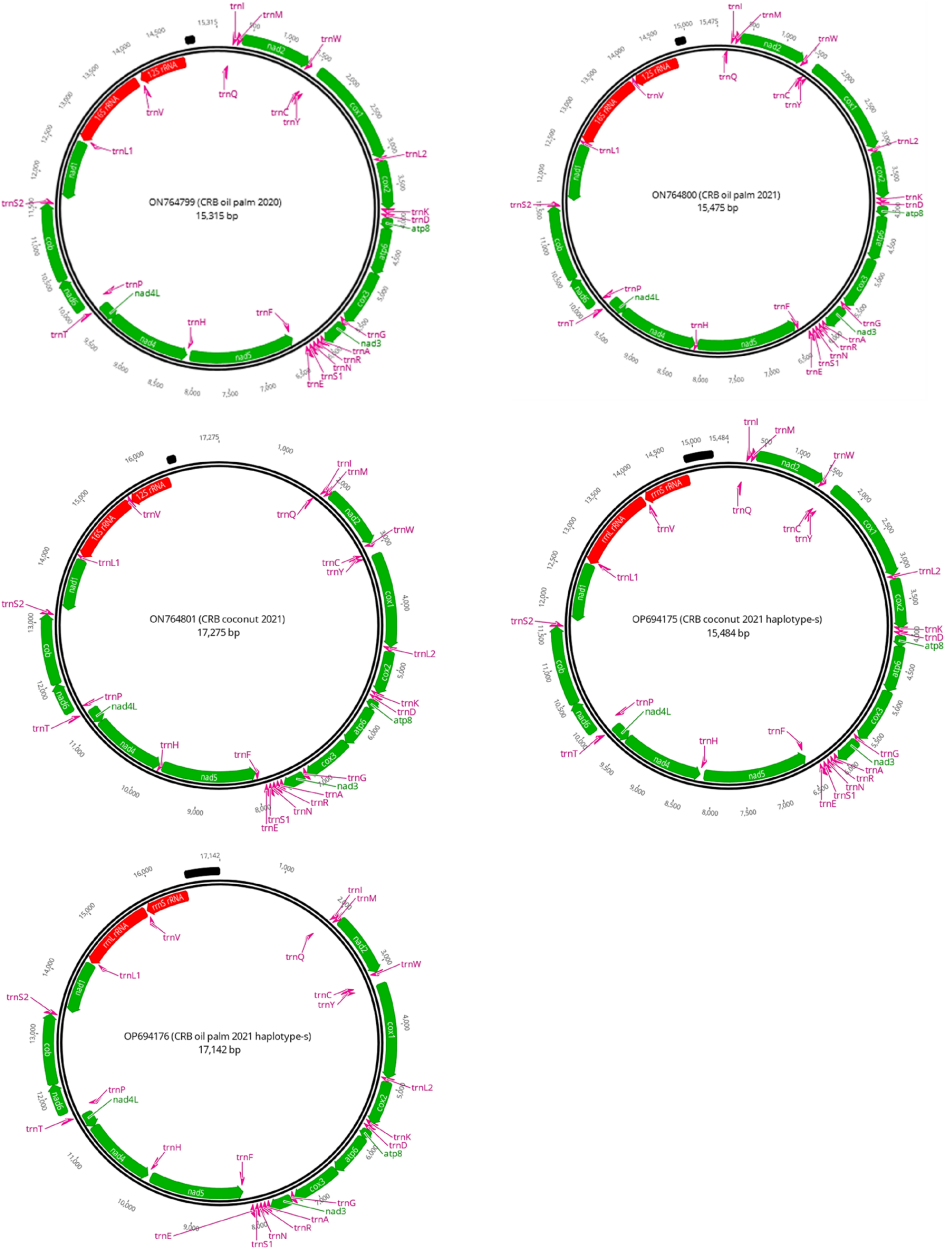
Circular map of the mitogenome of Johor CRB. The position and orientation of 13 PCG genes (green), 22 tRNA (pink), two rRNA genes (red), and the control region (black).

The mitogenome of all Johor CRBs collected in the present study featured a gene-packed section and a control region, also known as the D-loop region which contained components required for transcription and replication. It contained 13 PCGs, two rRNA genes, and 22 tRNA genes. Among the 13 PCGs, 9 PCGs (*nad2*, *cox1*, *cox2*, *atp8*, *atp6*, *cox3*, *nad3*, *nad6*, *cob*) were encoded in the majority strand (J strand) while 4 PCGs (*nad5*, *nad4*, *nad4L*, *nad1*) were encoded in the minority strand (N strand) (Fig. 2).

### Phylogenetic analysis

**Figure 3:**
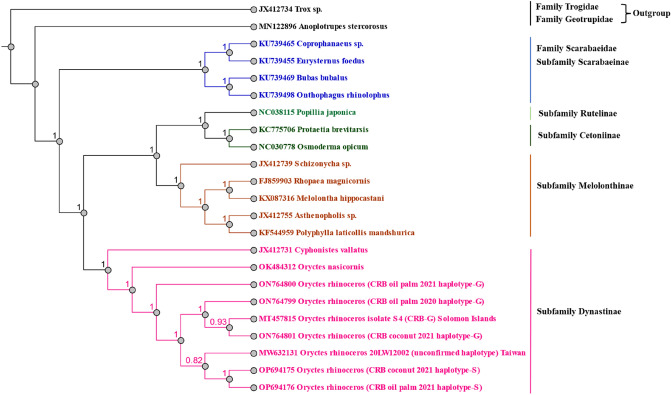
Bayesian inference of phylogenetic tree for *Scarabaeidae*, with the outgroups Family Trogidae and Geotrupidae, constructed using MrBayes plugin Genious prime version 2023.0.2^22^.

The phylogenetic analysis presented the relationship between the mitogenome of Johor CRBs and other members of the subfamily Dynastinae. The 23 datasets of mitogenomes of scarab beetles with Trogidae and Geotrupidae as the outgroup were aligned without removing redundant sequences or trimming end gaps from the alignment. The yielded alignment of the aligned mitogenomes sequence was 26,220 bp. Tree construction was inferred from Bayesian phylogenetic analysis using HKY85 model with an equal rate variation setting carried out in Geneious version 2023.0.2. Posterior probabilities were calculated over 2.0 × 10^6^ generations. The Bayesian tree showed the more robust phylogeny tree of scarab beetles which has successfully separated Family Scarabaeidae as one clade per subfamily with a posterior probability of 100% (Fig. 3).

The mitogenome of Johor CRBs was compared to the complete mitogenome of CRB-G from the Solomon Islands (GenBank accession number: MT457815). The percent of sequence identity of the mitogenomes of Johor CRBs (GenBank accession number: ON764799, ON764801, OP694175, and OP694176) was around 99% except the Johor CRB-G collected from the oil palm in 2021 (GenBank accession number: ON764800) was 89.24% (Table 8).

### OrNV confirmation and symptoms

**Figure 4:**
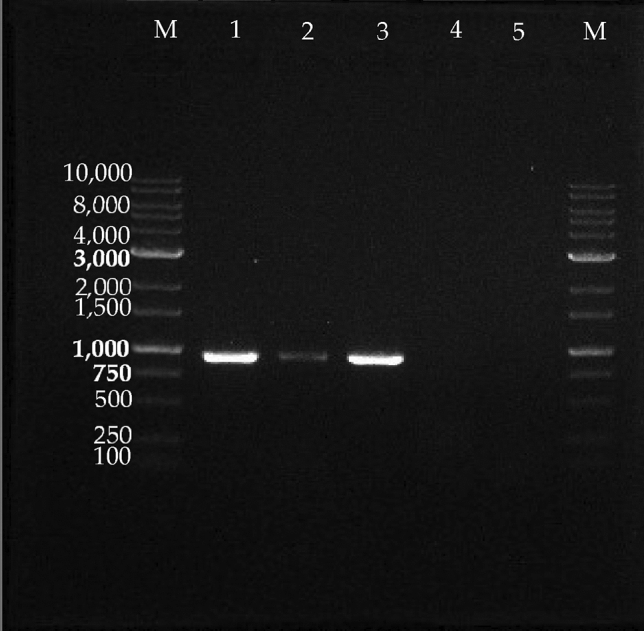
Uncropped and unadjusted images of gel. Images of agarose gel of gel electrophoresis of the DNA amplification product to detect OrNV in Johor CRB samples. M: CSL-MDNA 1 kb ladder, (1) oil palm Johor CRB-G 2020, (2) oil palm Johor CRB-G 2021, (3) coconut Johor CRB-G 2021, (4) coconut Johor CRB-S 2021, (5) oil palm Johor CRB-S 2021. The PCR product was run on 1% agarose gel in 1 × TAE buffer (w/v) added with DNA stain (Canvax, Brightmax) at 65 V for 40 min.

The gDNA of Johor CRB samples (n = 30) confirmed the presence of OrNV by PCR amplification (Fig. 4). The presence of OrNV in the Johor CRB-G samples collected from oil palm (n = 5) and coconut (n = 3) plantations was detected with a target band of 945 bp. However, the OrNV was not detected in the Johor CRB-S haplotype samples (n = 22).

**Figure 5:**
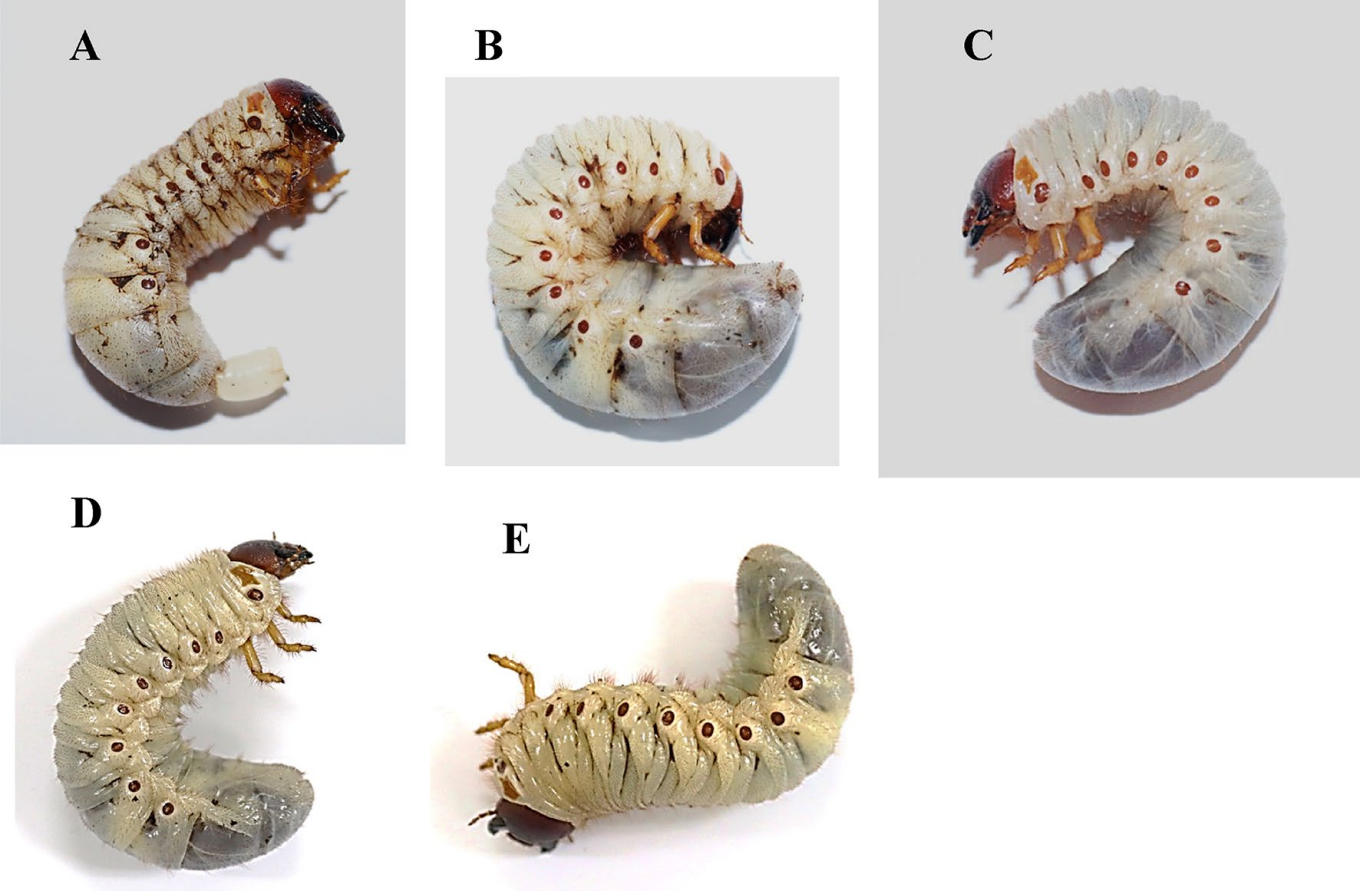
Symptoms of Johor CRB with OrNV infection. (**A**) Diseased oil palm Johor CRB-G 2020 with milky white abdomen and prolapsed rectum, (**B**) diseased oil palm Johor CRB-G 2021 with swollen milky white abdomen, (**C**) diseased coconut Johor CRB-G 2021 with swollen milky white abdomen but smaller in size, (**D**) coconut Johor CRB-S 2021 with negative OrNV infection, and (**E**) oil palm Johor CRB-S 2021 with negative OrNV infection.

The Johor CRB-G with positive OrNV detection (Fig. 5C) had a milky white body with bigger translucent abdomen than those Johor CRB-S with negative OrNV detection which had beige abdomen (Fig. 5D,E). The diseased oil palm Johor CRB-G 2020 exhibited prolapsed rectum in general (Fig. 5A) while those diseased oil palm Johor CRB-G collected in 2021 displayed a swollen abdomen without prolapsed rectum (Fig. 5B). The diseased coconut Johor CRB-G also exhibited similar symptoms to those of oil palm Johor CRB-G 2021 except the translucent abdomen was much smaller in size (Fig. 5C).

## Discussions

This study reported the mitochondrial genome of CRBs collected in oil palm and coconut plantations in Johor, Malaysia. Two different haplotypes, namely CRB-G and CRB-S, were discovered in similar breeding sites. It indicates an overlapping population of different haplotypes in one breeding site. These haplotypes have different length of mitogenome either within or between haplotypes. The mitogenome size of oil palm Johor CRB-G 2020 and 2021, coconut Johor CRB-G 2021, coconut Johor CRB-S 2021, oil palm Johor CRB-S 2021 was 15,315, 15,475, 17,275, 15,484 and 17,142 bp, respectively. The Johor CRB-G and CRB-S contained similar mitogenome size compared to CRB (unknown haplotype) from Taiwan (15,339 bp)^16^ but smaller than those CRB-G from Solomon Island (20,898 bp)^7^ and other Coleoptera species, *Protaetia brevitarsis* (20,319 bp)^23^, and *O. nasicornis* (20,396 bp)^24^. The difference in the mitogenome size are primarily due to the size variation of the non-coding region^25^. In general, the mitogenome has a non-coding region (NR) with AT-rich hairpin structures, tandem repetitions, and unusual patterns^26–28^. The largest NR of *O. rhinoceros* was identified as a putative control region (CR)^7^. Previous studies reported that the mitochondrial genome could be highly polymorphic even across individuals of the same species^29^.

The control region (CR) of Johor CRB-G and CRB-S contained extraordinarily high A + T composition which is often referred as “A + T-rich area” in insects^30^. This non-coding region involved in the initiation of mtDNA transcription and replication^31–33^. It demonstrates a high rate of nucleotide change, divergence of primary nucleotide sequences, and diverse fragment length between species and individuals^34^.

To date, the mitogenome of Johor CRB-S presented in this study is the first report of CRB haplotype S in the world. Both mitogenomes of the Johor CRB-G and CRB-S have a full feature of 37 genes: ATPase subunits 6 and 8 (*atp6* and *atp8*), cytochrome oxidase subunits 1 to 3 (*cox1-cox3*), cytochrome b (*cob*), NADH dehydrogenase subunits 1–6 and 4L (*nad1-6* and *nad4L*); small and large subunit rRNAs (*rrnL* and *rrnS*); and 22 transfer RNA (tRNA), which are the characteristics of metazoan mitogenomes^35,36^. Metazoan mitogenomes show diversity in several aspects, including length, tRNA secondary structure, gene order, the number and internal structure of regulatory areas, and sequence variation^35,37,38^. These characteristics can reveal the evolutionary links between species at high and low taxonomic levels^8^.

The mitogenomes of Johor CRBs contained standard gene order of insects, except for three tRNAs presenting the "tQ-tI-tM" order instead of the "tI-tQ-tM" order^8^. The *trnQ* gene precedes the *trnI* gene in the mitogenomes of Johor CRB collected from oil palm and coconut (Fig. 1). It is similar to the complete mitogenome of CRB from the Solomon Islands^7^ and Taiwan^16^. The *trnI* and *trnQ* genes were also found rearranged in the mitogenomes of all Hymenoptera species^39^ and were reported in flat bugs (Hemiptera, Aradidae)^40^. tRNA gene rearrangement had been observed in Lepidoptera and Neuropteran^14,41^. The tRNA rearrangement between the CR and *cox1* happened in Johor CRBs, and it has been proposed that this region may act as a "hotspot" for tRNA rearrangement^39^.

The mitogenomes of Johor CRB-G and CRB-S contained 13 PCGs with a regular initiation codon (ATN). A total of 10 PCGs ended with common stop codons (TAG or TAA) while three other genes, such as *atp6*, *cox3*, and *nad5* had an incomplete stop codon T, which is similar to the mitogenome of CRB-G from Solomon Islands^7^. Other lepidopteran mitogenomes featured incomplete stop codons, which are prevalent among their mitogenomes^42^.

Substitutions and indels in the PCGs indicate mutation in the mitogenomes. Based on the mauve alignment, substitutions and indels present in the mitogenome of Johor CRB-G and Johor CRB-S haplotypes, except the coconut Johor CRB-G. The coconut Johor CRB-S 2021 (OP694175) and oil palm Johor CRB-S 2021 (OP694176) have substitutions and indels found in 10 genes: *nad2, cox1, cox2, atp6, nad5, nad4, nad4L, nad6, cob,* and *nad1* genes when compared to all Johor CRB-G in this study and Solomon Islands. The Johor CRB-G contained substitutions and indels only in 6 genes: *nad5, nad4, nad4L, nad6, cob,* and *nad1*. Among the Johor CRB-G, the oil palm Johor CRB-G 2021 (GenBank accession number: ON764800) contained many substitutions in *nad5, nad4, nad4L, nad6, cob,* and *nad1* genes.

Mitochondrial DNA (mtDNA) genes such as *cox1* and *cox2* had been used in designing universal primers for DNA barcoding of invertebrates^43^. The presence of SNPs in *cox1* gene has been used to categorize the haplotype of CRB from Guam, Solomon Islands^5^. In Orthoptera, *cox2* gene was used to identify the orthopteroid insects^44^. In the present study, SNPs were detected in both the *cox1* and *cox2* genes of Johor CRB-G and Johor CRB-S. Four fixed base change was found in *cox1* gene, and one fixed base change was found in *cox2* gene that could possibly distinguish the Johor CRB-S group from the Johor CRB-G. For example, in *cox1* gene, the substitutions were located at nucleotide position 318 (G > A), 723 (T > C), 906 (C > T) and 1,080 (T > C) within the sequence fragments examined. The Johor CRB-G has more SNPs in *cox1* gene as compared to the partial *cox1* gene of CRB-G from Solomon Islands. An A > G transition at nucleotide position 426 was detected in the *cox2* gene of Johor CRB-G. In addition, the *nad2* and *atp6* genes showed 6 and 3 nucleotide substitutions in the Johor CRB-S group, respectively. In *nad2* gene, the substituitions were located at nucleotide position 333 (T > C), 591 (T > C), 642 (A > G) within the sequence fragments examined while in *atp6* gene, the substituitions were located at nucleotide position 64 (C > T), 207 (C > T), 542 (C > T) within the sequence fragments examined. The *cox1, cox2, nad2* and *atp6* genes were able to distinguish the Johor CRB-S from Johor CRB-G as well as the CRB-G from Solomon Islands.

The control region (CR) of Johor CRB-G and CRB-S contained extraordinarily high A + T composition which is often referred as "A + T-rich area" in insects^30^. This non-coding region involved in the initiation of mtDNA transcription and replication^31–33^. It demonstrates a high rate of nucleotide change, divergence of primary nucleotide sequences, and diverse fragment length between species and individuals^34^. The Johor CRB collected from the stump of coconut had a clear white body colour whereas the Johor CRB collected from decayed oil palm was white with a hint of light brown colour. Differences in the environment and food nutrition may influence a phenotypic change^45^. In general, the Johor CRB-G and Johor CRB-S were phenotypically similar. However, different haplotypes of Johor CRBs collected from the same sampling sites had exhibited different susceptibility towards OrNV infection. The CRB-G haplotypes collected from oil palm and coconut were confirmed positive to OrNV detection and infection. However, the CRB-S haplotype collected both from oil palm and coconut were confirmed negative to OrNV detection and infection. Even though Johor CRB-G and Johor CRB-S were found in the same sampling sites, only Johor CRB-G were susceptible to OrNV infection. The CRB-G and CRB-S from Johor Malaysia had exhibited different response to OrNV infection compared to those CRB-G and CRB-S reported in Pacific Islands^5^. This could be due to variation in the virulence of OrNV isolate from different geographical regions^46^. There were two OrNV strains, OrNV Kluang and OrNV Batu Pahat, were detected in Johor CRB-G^47^. The OrNV isolates found in Johor, Malaysia may have different virulence than those OrNV Solomon Islands isolate towards CRB-G.

Johor CRB-G exhibited different symptoms of OrNV infection. The oil palm CRB-G 2020 showed chronic lethal OrNV infection with swollen midgut and prolapsed rectum as reported in OrNV-infected CRBs^2,48,49^. In contrast, the oil palm CRB-G 2021 and coconut CRB-G 2021 did not have prolapsed rectum. Symptomatic infections were shown by the clinical signs and high level of viral particle production, to which the insect succumbs or survives depending on the state of its immune system^50^. OrNV-infected CRBs will exhibit a prolapsed rectum when they are severely infected^51^.

Melolonthinae, Cetoniinae, Dynastinae and Rutelinae were used in the phylogenetic analysis of scarabaeidae species. Previous study reported that the subfamily of Melolonthinae was paraphyletic while Cetoniinae was more closely linked to Dynastinae and Rutelinae^52–54^. The present study showed that the Dynastinae formed a monophyletic group as a clade while the Cetoniinae and Rutelinae formed sister clades that established a basal split with Melolonthinae. This result was similar to another previous study of two mitogenomes of scarab beetles^16^. However, our finding provides more robust support for branch nodes in which almost all branch nodes are equal to one. The Dynastinae, Cetoniinae, Rutelinae, and Melolonthinae are phytophagous group while the Scarabaeinae are coprophagous group^52,53^. The present Bayesian tree has successfully confirmed the correlation of the subfamily to their feeding habits.

The phylogenetic analysis has confirmed the oil palm Johor CRB-G 2020 (ON764799), the coconut Johor CRB-G 2021 (ON764801) and the CRB-G from Solomon Islands (MT457815) were monophyletic. On the other hand, the oil palm Johor CRB-S (OP694175), coconut Johor CRB-S (OP694176) and CRB from Taiwan (unconfirmed haplotype: NC059756) had a common ancestor. The oil palm Johor CRB-G 2021 (ON764800) revealed a separate ancestor from other Johor CRB-Gs. Although the BLAST result of the oil palm Johor CRB-G 2021 revealed a low (89.24%) sequence homology, it was grouped with other Johor CRB-Gs by in silico digestion. This indicates the oil palm Johor CRB-G 2021 has a unique mitogenome of CRB-G and is considered as an unrecognized haplotype of CRB-G.

## Conclusions

Two haplotypes of CRB were discovered in the oil palms and wild coconut in Johor, Malaysia. Both haplotypes can be found in the same sampling sites in the field. The Johor CRB-G samples were prone to OrNV infection while the Johor CRB-S were resistant to OrNV infection. The mitogenome of Johor CRBs was variable in the natural population due to its elevated mutation rate. Substitutions and indels in *cox1, cox2, nad2* and *atp6* genes were able to distinguish the Johor CRBs into two haplotypes. Further investigation is needed to study the relationship between the two haplotypes of Johor CRB and OrNV infections in the field.

## Materials and methods

### Ethics statement

No specific permits were required for the insect specimen collection in this study. All experiments were performed in accordance with relevant named guidelines and regulations. All sequenced insects are common species in Malaysia and are not included in the “Red List of Mammals for Peninsular, Malaysia version 2.0.

### Sample collection

Oil palm CRB-G 2020 (GPS coordinate: 2.0248117446899414, 103.25872039794922) and oil palm CRB-G and CRB-S 2021 (GPS Coordinate 2,0,310,530, 103,2,703,850) were collected from decayed palms in a private oil palm plantation in Kluang, Johor. Coconut CRB -G and CRB-S 2021 were collected from wild coconut trees in Batu Pahat, Johor (GPS Coordinate: 1.720853, 103.053085). The distance between the oil palm and the coconut sampling location was more than 50 km. The field studies did not involve endangered or protected species. 3rd instar larvae were extracted at Laboratory of Insect Pathology, Department of Plant Protection, Universiti Putra Malaysia, Serdang, Selangor.

### DNA extraction of CRB

Insect gut tissue was cut and washed with two times diluted 1 × PBS. The gut tissue was subjected to DNA extraction using a modified protocol of NucleoBond® RNA Soil (MachereyNagel GmbH & Co., Germany). Briefly, approximately 1–1.5 g sample was suspended in 3.2 ml Lysis Buffer E1 and divided into four portions. Each portion (~ 800 µl) was transferred into a 2 ml NucleoSpin® Bead Tubes Type A. 100 µl of buffer OPT was added to the mixture, followed by 100 µl of phenol: chloroform: isoamyl alcohol (25:24:1 v/v). The sample was lysed by bead beating for 5 min at 2280 rpm on a mechanical cell disruptor. The sample tubes were then centrifuged for 2 min at 14,800 rpm. The supernatant of different tubes was pooled into a 15 ml centrifuge tube to a final volume of 2.5 ml. An aliquot of 313 µl of binding Buffer E2 was added, and the tube was inverted five times, then incubated for 2 min at room temperature. The tube was then centrifuged for 2 min at 6000 rpm. The supernatant was transferred into a NucleoBond® RNA Column (including a filter) pre-equilibrated with 12 ml of equilibration Buffer EQU. The supernatant was loaded into the centre of the filter. The filter was washed with 6 ml of Buffer E3; the flow through and filter were then discarded. The NucleoBond® RNA Column without a filter was washed with 8 ml of Buffer E4. The column was transferred to a fresh 50 ml tube, and the DNA was eluted with 5 ml of elution buffer EDNA. The first eluted DNA was mixed with 3.5 ml of isopropanol. The mixture was then loaded into a NucleoSpin® Finisher Column and centrifuged for 2 min at 6000 rpm. The column was washed with 1 ml Buffer E5, followed by drying using centrifugation at 6000 rpm for 2 min. Finally, the DNA was eluted with 100 µl of RNase-free H_2_O. DNA was subjected to RNase treatment at 37 °C for 30 min and then precipitated with phenol: chloroform: isoamyl alcohol extraction, followed by ethanol precipitation. Lastly, the DNA pellet was dissolved in 50 µl of RNase-free H_2_O.

### DNA quality check

The quality of the DNA samples was confirmed prior to Next-generation sequencing (Supplementary Table 1). Two methods in quality control of DNA samples were used. Method 1: DNA degradation and potential contamination was assessed on 1% agarose gel. Method 2: the DNA concentration was determined using a Qubit® 2.0 Fluorometer and the Qubit® dsDNA Assay Kit (Life Technologies, CA, USA). The sample with OD values between 1.8 and 2.0, and DNA concentration greater than one µg was used to construct a library. The samples were sent for Next-generation sequencing using the Illumina platform at Novogene Co., Ltd. Singapore.

### Library construction

A total of 1 µg of DNA sample was used as input material for library preparation. Libraries were generated using the NEBNext® Ultra™ D.N.A. Library Prep Kit (NEB, USA). The index codes were added to attribute sequences to each sample. The DNA sample was fragmented by sonication to a size of 350 bp. Then, the DNA fragments were end-polished, A-tailed, and ligated with the full-length adaptor for Illumina sequencing with further PCR amplification. Finally, the PCR products were purified (AMPure XP system), and libraries were analyzed for size distribution by Agilent 2100 Bioanalyzer and quantified using real-time PCR.

### Illumina sequencing

The clustering of the index-coded samples was performed using cBot Cluster Generation System. After cluster generation, the library preparation was sequenced on an Illumina NovaSeq6000 platform, and paired-end reads were generated.

### Mitogenome assembly, annotation, and analysis

The quality of raw reads was inspected with FastQC v.0.11.9^55^. Low-quality reads (Q ≤ 28) were removed with fastp v.0.20.1^56^. The mitogenome was assembled with MitoZ v2.4-alpha^57^. Mitogenome annotation was performed with the Mitos2 web server (http://mitos2.bioinf.uni-leipzig.de/index.py) with parameters as follows: Reference: "RefSeq 89 Metazoa" and genetic code: "5 Invertebrate". The contig was imported to Geneious prime version 2023.0.2, and the mitogenomes were finally visualized with the Geneious prime version 2023.0.2.

### In silico digestion

The assembled *cox1* gene sequences were aligned with the CRB *cox1* gene (526 bp) obtained from the GenBank using the MAFFT alignment with the default setting parameters in Geneious Prime version 2023.0.2. The alignment was further trimmed to reduce gaps, yielding a 526-bp sequencing fragment. The trimmed sequence was cut with *Mse*I restriction enzyme and RFLP pattern was analysed for confirmation of CRB haplotypes.

### Phylogenetic analysis

The phylogenetic tree was constructed with additional taxa (complete or partially complete mitogenome data) available at the NCBI (Table 9). Sixteen species from five Scarabaeidae subfamilies (Dynastinae, Rutelinae, Cetoniine, Melolonthinae, and Scarabaeinae) and outgroups from the superfamily of Scarabaeoidea (Family Trogidae and Geotrupidae) were compared. Each mitochondrial genome was aligned using MAFFT^58,59^ with default parameter settings in Geneious Prime version 2023.0.2. (https://www.geneious.com). The phylogenetic tree construction was inferred from Bayesian phylogenetic analysis using the HKY85 substitution model with an equal variation setting carried out in Geneious Prime version 2023.0.2 (https://www.geneious.com). The posterior probability was calculated with a 1,000,000-chain length and burn-in length of 100,000 using molecular clock computation with uniform branch length gamma 1 to 1,000,000.

**Table 9:** Taxa with complete or partial mitogenome sequences used for the phylogenetic analysis.

Accession	Organism	Genome type	Missing genes	Contains control region	Sequence length (bp)	Reference
FJ859903	*Rhopaea magnicornis*	Complete	None	Yes	17,522	^41^
JX412731	*Cyphonistes vallatus*	Partial	nd1	No	11,629	^60^
JX412734	*Trox* sp.	Partial	nd2; cox1	No	11,622	^60^
JX412739	*Schizonycha* sp*.*	Partial	nd2	No	13,542	^60^
JX412755	*Asthenopholis* sp*.*	Partial	nd2	No	12,352	^60^
KC775706	*Protaetia brevitarsis*	Complete	None	Yes	20,319	^23^
KF544959	*Polyphylla laticollis mandshurica*	Partial	None	No	14,473	^61^
KU739455	*Eurysternus foedus*	Partial	None	No	15,366	^62^
KU739465	*Coprophanaeus* sp*.*	Partial	None	No	15,554	^62^
KU739469	*Bubas bubalus*	Partial	None	No	16,035	^62^
KU739498	*Onthophagus rhinolophus*	Partial	None	No	16,035	^62^
KX087316	*Melolontha hippocastani*	Partial	None	No	15,485	Unpublished
MN122896	*Anoplotrupes stercorosus*	Partial	nd2	No	13,745	Unpublished
NC030778	*Osmoderma opicum*	Complete	None	Yes	15,341	^63^
NC038115	*Popillia japonica*	Complete	None	Yes	16,541	^12^
MT457815	*Oryctes rhinoceros* isolate 4	Complete	None	Yes	20,898	^7^
NC059756	*Oryctes rhinoceros* voucher 20LW12002	Complete	None	Yes	15,339	^16^
OK484312	*Oryctes nasicornis*	Complete	None	Yes	20,396	^24^

### Confirmation of OrNV infection

Briefly, a total 25 µl PCR reaction mixture was prepared by mixing 12.5 µl of PCR GoTaq® Green Master Mix, 2.5 µl of the forward and reverse primer, 2.5 µl of DNA template, and 5 µl autoclaved distilled water. The primers of OrV15^64^ was used for the OrNV confirmation. The PCR diagnosis was carried out under the following conditions: an initial denaturation of 95 °C for 2 min, and 35 cycles of denaturation at 95 °C for 30 s, annealing 50 °C for 45 s, and extension 72 °C for 1 min with a final extension at 72 °C for 5 min. Amplified DNA samples were run on 1% agarose gel prepared in 1 × TAE buffer at 68 V for 40 min.

### Supplementary Information

Supplementary Table 1.

## Data Availability

The assembled data are available on the website of NCBI with accession numbers: ON764799, ON764800, ON764801, OP694175, and OP 694176.
