# Supplementary material for: Variants in the mitochondrial genome sequence of Oryctes rhinoceros (Coleoptera: Scarabaeidae) infected with Oryctes rhinoceros nudivirus in oil palm and coconut plantations
Source: Sci Rep. 2023 Oct 6;13:16850. doi: 10.1038/s41598-023-43691-w (PMC10558481; doi:10.1038/s41598-023-43691-w)
Supplement: Supplementary file 1 — Supplementary Table 1. [file 41598_2023_43691_MOESM1_ESM.docx]

**Manuscript ID: 704d89cc-7779-407b-8436-bdfbc5a985af**

**Variants in the Mitochondrial Genome Sequence of *Oryctes rhinoceros* (Coleoptera: Scarabaeidae) Infected with *Oryctes Nudivirus* in Oil Palm and Coconut Plantations**

Table 1: Quantity of DNA samples for next generation sequencing (NGS)

| **Samples ID** | **A260/280** | **A260/230** | **Nanodrop Spectrophotometer** | **Qubit Fluorometer** |
| --- | --- | --- | --- | --- |
|  |  |  | **Concentration (ng/µl)** | **Concentration (ng/µl)** |
| Oil palm Johor CRB-G 2020 | 1.77 | 1.47 | 41.45 | 42 |
| Oil palm Johor CRB-G 2021 | 1.96 | 2.23 | 672.1 | 480 |
| Oil palm Johor CRB-S 2021 | 1.91 | 2.02 | 520.15 | 424 |
| Coconut Johor CRB-G 2021 | 1.94 | 2.56 | 219.25 | 148 |
| Coconut Johor CRB-S 2021 | 1.77 | 1.39 | 151.6 | 104 |
